# Deuterated SiNx: a low-loss, back-end CMOS-compatible platform for nonlinear integrated optics

**DOI:** 10.1515/nanoph-2022-0626

**Published:** 2023-02-13

**Authors:** Xavier X. Chia, Dawn T. H. Tan

**Affiliations:** Photonics Devices and Systems Group, Engineering Product Development, Singapore University of Technology and Design, 8 Somapah Road 487372, Singapore, Singapore; Agency for Science, Technology and Research (A*STAR), Institute of Microelectronics, 2 Fusionopolis Way 138634, Singapore, Singapore

**Keywords:** chemical vapour deposition, nonlinear optics, photonic-integrated circuits, silicon photonics

## Abstract

Silicon nitride (SiN) has surged into prominence as a material for photonic-integrated circuits (PICs) in the past decade, well regarded for its broadband transparency, compatibility with complementary metal oxide semiconductor (CMOS) fabrication processes and high optical bandgap that avoids two-photon absorption. However, current fabrication methods result in users having to choose between low thermal budgets and low losses, which are suboptimal given that both are necessary to facilitate a wide range of applications. Recently, works have emerged featuring PICs fabricated using deuterated silicon nitride (SiNx:D) – SiNx films grown using deuterated precursors instead of conventional hydrogenated ones. This decreases material absorption near the telecommunications bands at 1.55 µm previously present due to parasitic silicon–hydrogen and nitrogen–hydrogen bonds, attaining low-loss PICs realised using a low temperature, back-end-of-line CMOS-compatible fabrication plasma-enhanced chemical vapour deposition process. These devices have shown promise for both linear and nonlinear applications and the platform has the potential to be instrumental in realising highly efficient chips with co-packaged electronics and photonics devices. This paper reviews recent developments on the SiNx:D platform and provides a glance at future advancements for this highly promising material.

## 1 Introduction and background

In the past decade of research in photonic-integrated circuits (PICs), silicon nitride (SiN) thin films have been a dominant material for the realisation of efficient passive devices. The applications of these films are ubiquitous – the material has been previously employed in areas such as the passivation of solar cells [1, 2], microstructures [3, 4] and membranes [5, 6] in micro-electromechanical systems (MEMs) long before their rise in popularity for C-band integrated optics. Over time, low-pressure and plasma-enhanced chemical vapour deposition (LPCVD/PECVD) methods have become fabrication methods of choice for depositing SiN thin films given their consistency and customizability, and it has become rare to see other methods used in their fabrication although some of these such as reactive [7] or magnetron sputtering [8] have emerged recently.

The study of the nonlinear optical properties of silicon nitride dates back to 2008, when Ikeda et al. investigated the thermal and Kerr nonlinearity in PECVD-grown SiN films and their use in photonic waveguides [9, 10]. Since then, optical devices implemented on silicon nitride have achieved a remarkable number of milestones over the years, most significant of which is their impact on microresonator frequency combs [11–20], spanning applications such as high-speed communications [21–25] and metrology [26–30]. The strength of the silicon nitride (SiN) platform lies in its broadband transparency and material compatibility with modern foundry processes [31], which has made SiN a popular platform for integrated optics with a growing number of groups advancing SiN technologies. Of note, the ultra-low losses achievable in LPCVD-grown silicon nitride that are thermally annealed at temperatures of up to 1200 °C have yielded ultra-low losses as low as sub-dB/m [32–37], instrumental to the observations of nonlinear optical phenomena at low powers.

Aside from the study of SiN that is close to its stoichiometric composition for nonlinear optics, a separate effort in the study of tailoring the linear and nonlinear properties of SiNx films was taking place. Chemical vapour deposition techniques for growing SiNx films provide a design degree of freedom for compositionally tailoring SiNx films between silicon-rich or nitrogen-rich chemistries, effectively providing a sliding scale in the films’ optical properties. In the fabrication of CVD silicon-nitride films, the Si:N ratios may be varied by controlling the ratios of the precursor gases used to provide the Si and N content in the films. Some common precursor gases used for supplying Si content include SiH_4_ and SiH_2_Cl_2_, whereas precursor gases used to supply the N content include NH_3_ and N_2_ gas. It has been documented by various groups that tuning the precursor gas ratio to have a higher Si precursor gas flow rate may lead to silicon-rich nitride films with a higher linear and nonlinear refractive index [38–51]. In general, work in this area has aimed to achieve a maximum Kerr nonlinearity while retaining a sufficiently large material bandgap to preclude two-photon absorption at the telecommunications wavelength. For clarity, the abbreviation SiN shall henceforth be used to refer to films with Si:N ratios close to stoichiometric values (Si_3_N_4_), while SiNx will be used to refer to SiN and SRN in general.

In the domain of nonlinear optics, SiNx also possesses an advantage over silicon-on-insulator technologies through the avoidance of two-photon absorption (TPA) at telecommunications wavelengths due to their larger bandgap, which can range from 2.1 eV (Si_7_N_3_) [52] to 5 eV (Si_3_N_4_) depending on the Si:N ratio. In PECVD silicon-rich nitride waveguides, Kerr nonlinearity above that of crystalline silicon devices have also been achieved while retaining a bandgap above the TPA threshold [47], which evidences the suitability of silicon-rich nitride as a more viable candidate than silicon in nonlinear integrated optics.

However, certain important drawbacks associated with CVD SiNx platforms limit the platform from becoming an ideal material. During deposition, silane (SiH_4_) or dichlorosilane (SiH_2_Cl_2_ or DCS) is used as precursor gases to introduce silicon (Si) and nitrogen (N_2_) or ammonia gas (NH_3_) for the nitrogen component, which results in the introduction of hydrogen content in the chamber. These hydrogen atoms are incorporated into the film, forming parasitic Si–H and N–H bonds that have infrared vibrational modes in regions around 2157 cm^−1^ to 2250 cm^−1^ and 3290 cm^−1^ to 3464 cm^−1^, respectively [53–55]. While these do not directly impede the propagation of light in the C-bands, they result in absorption overtones that peak near 1.52 µm and bleed into the region at the telecommunications bands near 1.55 µm. This absorption mechanism has been documented to limit the propagation losses in SiNx waveguides to roughly 1.5 dB/cm [44, 56]. This issue also tends to affect PECVD films more extensively as the low process temperature (ranging from 270 °C to 350 °C) results in considerable hydrogen bonds becoming incorporated within the films [57]. Some degree of success in reducing N–H related absorption has been achieved by replacing ammonia gas (NH_3_) with nitrogen (N_2_), but the remaining hydrogen content from Si–H bonds continues to cause deleterious losses at the important telecommunications wavelengths [58].

In response to SiNx material absorption from hydrogen containing bonds, the silicon photonics community has largely responded through the use of LPCVD deposition and high temperature annealing steps that eliminate the parasitic bonds in a high temperature environment of up to 1200 °C, such as in the photonic Damascene process [59, 60] or other related subtractive methods [35, 61]. While this has resulted in great strides in the improvement of silicon photonics technologies, the high temperatures make them less suitable for back-end-of-line (BEOL) complementary metal-oxide semiconductor (CMOS) requirements, which tend to have a thermal budget between 400 °C and 525 °C. The manufacturing of commercial silicon photonics that include application specific integrated circuits include electrical interconnects in the lower layers, defined by many front-end-of-line (FEOL) processes. This is followed by low k- or copper-based metal interconnects followed by a photonics layer at the top. By this point in the process, any high temperature processes [62–64] employed to process the photonics layer at the top will negatively affect the electrical device layers defined on the front end, including dopant migration and melting of copper, which makes LPCVD silicon nitride that has a high thermal budget incompatible with BEOL processes.

**Figure 1:**
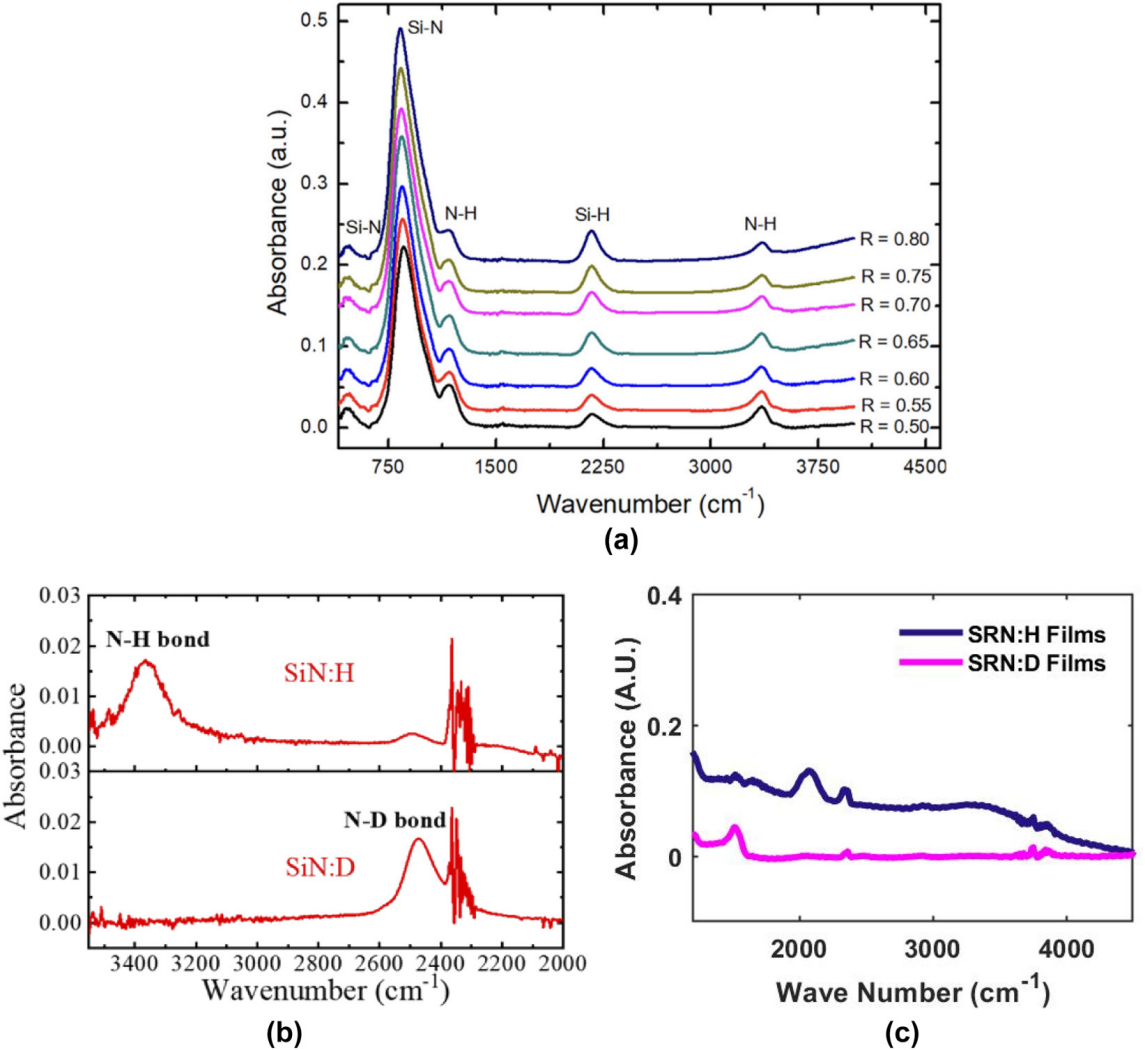
Fourier Transform Infrared (FTIR) spectra from various SiNx films in literature, showing infrared vibrational modes of constituent bonds. (a) FTIR data of PECVD SiNx:H films deposited with varying gas ratios. *R* is given as the ratio of SiH_4_:(SiH_4_ + NH_3_) used during deposition. Peaks corresponding to infrared vibrational modes of specific modes highlighted in the film, approximately located at 460 cm^−1^, 840 cm^−1^, 1170 cm^−1^, 2160 cm^−1^ and 3360 cm^−1^ corresponding to Si–N symmetric stretching, Si–N asymmetric stretching, N–H wagging, Si–H stretching and N–H stretching modes, respectively. Figure adapted from [53]. (b) FTIR spectrum for SiNx:H and SiNx:D films, showing the absorbance locations associated with the N–H and N–D bonds [67]. (c) FTIR absorbance curve of PECVD SRN:D film versus SRN:H film. Bond regions where Si–H and N–H curves are expected noticeably empty in the SRN:D curves due to the lack of hydrogen atoms in the precursor gases used during deposition. Figure adapted from [51, 65].

More recently (as is the subject of this mini-review), we have seen an increase in popularity of deuterated silicon nitride (SiNx:D) – silicon nitride films deposited using deuterated silane (SiD_4_) and N_2_ gas, which removes hydrogen from the precursor gases entirely, importantly while maintaining low deposition temperatures below 400 °C. This has the effect of completely replacing Si–H and N–H bonds with Si–D and N–D bonds, which results in a change in infrared vibrational modes of the film by virtue of the isotopic shift. This change has profound implications, for it shifts the Si–H related bond absorption overtone at the 1.52 μm region to 2.1 μm (associated with Si–D), completely away from the entire telecommunications band. Figure 1(b) and (c) show the infrared vibrational spectra obtained using Fourier-transform infrared spectroscopy (FTIR) of SiNx:H versus SiNx:D films, demonstrating the effects of substituting deuterium for hydrogen in the precursor gases. The mechanics of this phenomenon arise from changes in the vibrational bond energies [65], which are described using the quantum harmonic oscillator model and allow us to predict the shift in the absorption overtones [66]:

(1) $$-\frac{h^{2}}{2m}\frac{d^{2}\psi }{dx^{2}}+\frac{1}{2}kx^{2}\psi =E\psi$$

Here, *h* is Planck’s constant, *Ψ* is the wave function, *x* is the displacement from equilibrium and *E* is energy. For the boundary conditions of *Ψ* = 0, *x* = ±∞, this generates the closed form expression for the vibrational bond energy as given by [26]:

(2) $$E_{\upsilon }=h\cdot \left(1/2\pi \right).\left(\upsilon +1/2\right)\sqrt{k/m_{1}+k/m_{2}}$$

where *υ* is the vibrational quantum number (an integer), *k* is the bond’s force constant and *m*_1,2_ is the atomic mass for the two atoms making up the bond. From Eq. (2), it is observed that variations in *m*_1_ or *m*_2_ will result in a change in the vibrational bond energy. In the case of SiNx:D, hydrogen atoms are substituted with deuterium, which retains the force constant of the bond while increasing the molecular mass, given that deuterium and hydrogen have an atomic mass of approximately 2 and 1, respectively. A substitution of deuterium for hydrogen thus gives rise to the shift in molecular absorption spectra also known as the isotope shift that pushes absorption regions away from the telecommunications bands near 1.55 µm [58].

The resulting SiNx:D devices achieve losses and resonator quality factors equivalent to unannealed LPCVD films [65, 68] and improvements in propagation losses across the telecommunications bands [56] while retaining back-end CMOS compatibility due to process temperatures. The PECVD-deposited films also have the advantage of a single-step deposition process not privy to LPCVD deposition methods that require additional process steps to circumvent high film stress such as the scoring of trenches or a multi-stage deposition process. Notably, LPCVD grown SiN films typically have high stress on the order of 1 GPa [48, 60] while a 568 nm thick ICPCVD SiN:D film was characterized by Chiles et al. [69] to possess film stress of 191 MPa.

The advantage of SiNx:D lies in that it combines the advantages of PECVD and LPCVD SiNx:H, yielding films with high refractive index, high nonlinearity, low losses and low film stress in a convenient single-step deposition method. This circumvents the strict trade-off of low losses or CMOS compatibility that users of the SiNx platform have had to reckon with thus far. Since their introduction in 2017 [56], SiNx:D PICs have experienced intensified interest, being involved in a number of works including ultra-low-loss CMOS-compatible waveguiding devices, frequency comb generation and soliton generation. In the subsequent sections, we will cover recent advancements in SiNx:D devices in both the linear and nonlinear domains. The work then concludes with a brief section on the future outlook for these devices.

## 2 Recent work in linear optics

The first effective demonstration of SiN:D waveguides was presented by Hiraki et al. in 2017 [56]. In this work, an SiN:D film was fabricated via electron-cyclotron-resonance PECVD (ECR-PECVD) at 200 °C using SiD_4_ and N_2_ gas as the precursor gases. The resulting film possessed a refractive index of 1.87 at 1550 nm and 1.8 cm long waveguides with a cross section of 0.55 µm × 1.1 µm (height × width) fabricated on the film showed a TE_00_ mode propagation loss of 0.47 dB/cm. Reference SiN:H films fabricated using the same deposition process yielded hydrogen contents almost 20 times higher than the SiN:D film, evidencing the effectiveness of hydrogen reduction in improving material absorption losses. When benchmarked against devices fabricated on SiN:H films, the SiN:D devices presented significant improvements in transmission in the wavelength range from 1.4 µm to 1.6 µm.

**Figure 2:**
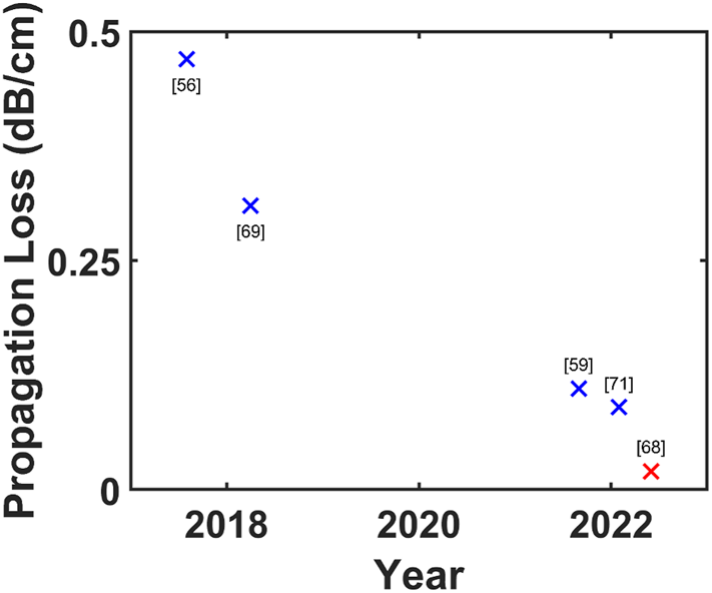
Table showing progressive improvements in propagation loss performance for SiN:D devices reported in literature. Blue crosses (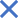) indicate loss values achieved with devices using high modal confinement while red crosses (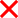) denote low modal confinement where significant amounts of power of the propagating mode is located within the cladding. In general, waveguide geometries with lower modal confinement have lower susceptibility to sidewall roughness and associated losses, while high modal confinement geometries exhibit stronger nonlinear properties.

The use of SiN:D as an optical platform has gained significant traction since then, inspiring a number of works [67, 69–71] that leverage the advantages afforded by the material and fabrication process. In contrast with low-loss LPCVD SiNx:H waveguides that required multi-stage processes including deposition, the use of trenches to alleviate high stress and high temperature annealing, advancements in SiNx:D research have yielded devices with increasingly lower losses that can be realised using a simple single-stage deposition (Figure 2). Micro-ring resonators (MRRs) with intrinsic quality factors up to 1.2 million [72] and devices with losses as low as 0.17 dB/cm [67] have since been demonstrated. In these works, the SiN:D films were deposited via Inductively-Coupled Plasma CVD (ICPCVD – a high-density plasma variant of PECVD) at 270 °C, resulting in films with a refractive index of ∼1.99 at 1550 nm and a film thickness of 880 nm. The resulting devices were ideal candidates for frequency comb generation given the relatively higher nonlinearity compared to silicon-dioxide and anomalous dispersion afforded by the thick SiN:D layer and will be covered more in-depth in the following section on nonlinear optics.

**Figure 3:**
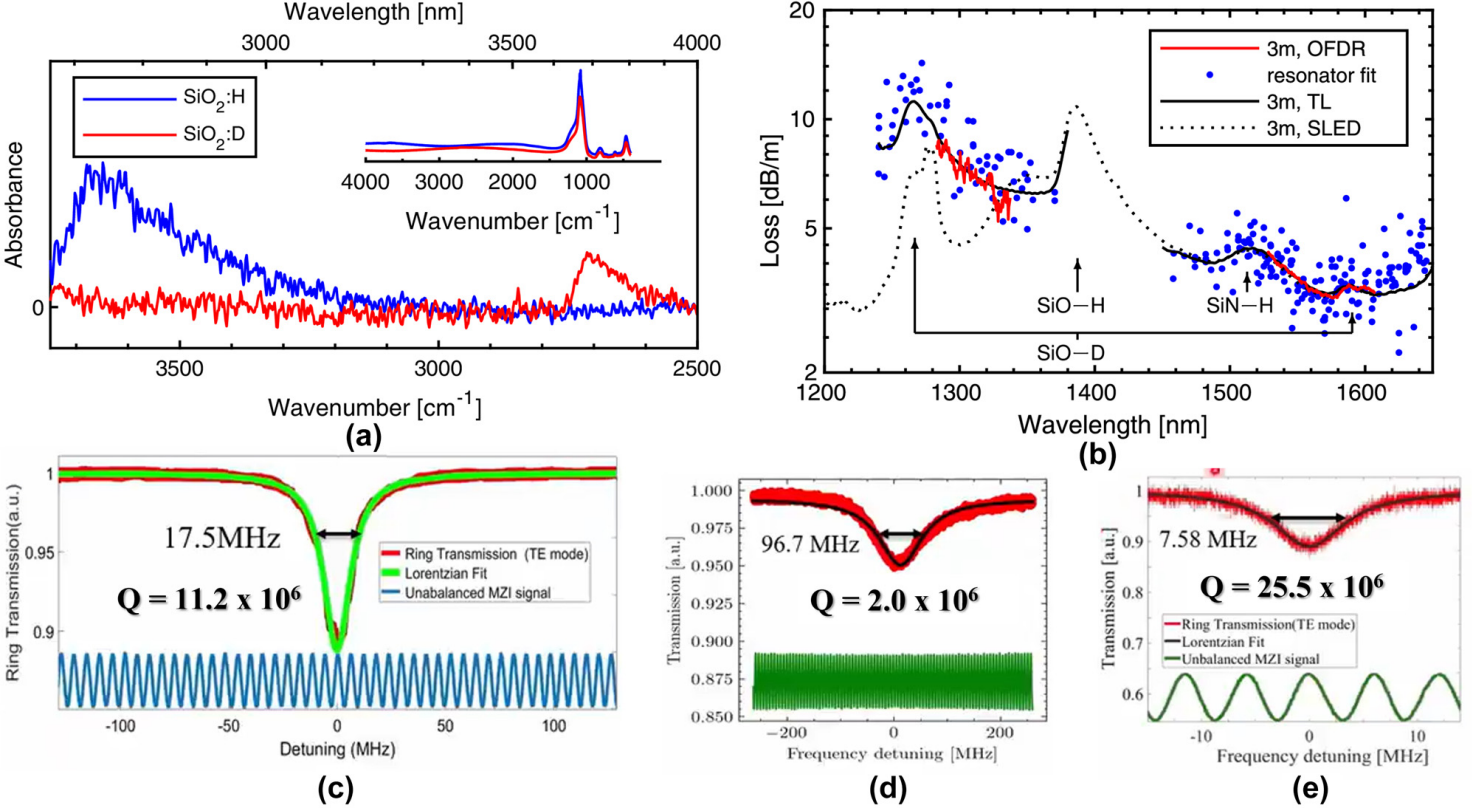
FTIR measured absorbance of SiO_2_:H and SiO_2_:D thin films, which results in a shift in the fundamental SiO–H vibrational mode at 2.73–3.74 µm for SiO–D. The inset shows the FTIR spectrum in an extended range. (b) Measured waveguide losses for SiN waveguides with SiH_4_-grown and SiD_4_ grown SiO_2_ cladding. The losses were measured using super luminescent light emitting diode transmission measurements and OFDR measurements. Measured MRR resonator transmission and loaded quality factor for (c) SiD_4_ grown SiN core, (d) 800 °C LPCVD SiN core, (e) 800 °C LPCVD SiN core annealed at 1050 °C for 7 h. MRRs in (c), (d) and (e) used an SiO_2_ cladding grown using SiD_4_. (a) and (b) are from Ref. [73]. (c)–(e) are from Ref. [68].

Propagation losses in waveguides can be further reduced through the use of SiD_4_ gas in the fabrication of the SiO_2_ cladding, forming a waveguide with a SiN core and SiO_2_ cladding grown entirely with precursor gases containing deuterium instead of hydrogen (Figure 2). Jin et al. documented a comparison between SiO_2_ thin films grown using SiH_4_ and SiD_4_ [73]. The use of SiD_4_ gas instead of SiH_4_ shifts the first absorption overtone of SiO–H from 1390 nm to 1870 nm in SiO–D, well outside the telecommunications band. Figure 3(a) shows the measured absorbance for both films, obtained using Fourier transform infrared spectroscopy. It is observed that the deuterated SiO_2_ films possess significantly lower absorbance for wavenumbers between 3300 and 3700 cm^−1^, which correspond to the fundamental SiO–H absorption. Conversely, higher absorbance is observed for wavenumbers between 2600 and 2700 cm^−1^, corresponding to the fundamental SiO–D absorbance. The use of deuterated SiO_2_ cladding is of particular importance for waveguide devices, which are weakly confined (and equivalently have a large effective area), as a significant part of the electric field would reside in the cladding and be subjected to attenuation from material losses. At the same time, low confinement waveguide geometries (such as those shown in columns 1 and 3 of Table 1) are an excellent approach to achieving extremely low-loss waveguides due to lowered susceptibility to sidewall roughness and its associated losses. In such waveguides, the evanescent tails of the mode extend considerably into the cladding, and the interaction of the mode with the waveguide sidewalls that are subjected to fabrication induced roughness will be very small. This limits the impacts of scattering-related losses in the propagating modes. Such waveguides were first studied in non-deuterated, LPCVD grown silicon nitride, cladded with deuterated SiO_2_ cladding. Around 8–9 dB/m losses in waveguides with a width and height of 2.8 μm and 80 nm–100 nm, respectively, were achieved [32]. These were then extended to similarly low confinement waveguides incorporating deuterated silicon dioxide cladding, where losses in similarly sized waveguides were demonstrated to decrease to 5 dB/m [73]. It may be observed from Figure 3(b) that a 30-fold loss reduction is enabled by the deuterated precursor gas at a wavelength of 1400 nm (300 dB/m to 10 dB/m), whereas optical frequency domain reflectometry measurements reveal a reduction in the propagation losses from 8 dB/m to 3 dB/m at 1550 nm.

**Table 1:** Properties of SiNx films reported in literature.

Parameters	SiNx:H		SiNx:D
Deposition type	LPCVD	PECVD	PECVD
Lowest recorded propagation loss @ 1.55 µm (waveguide dimensions in W × H, propagating mode)	Very low (annealed) 0.034 dB/m [74] (11 µm × 800 nm, TM mode)	Fairly high 1.5 dB/cm [44] (1.5 µm × 300 nm, TE mode)	Low 2.0 dB/m [68] (6.0 µm × 80 nm, TM mode)
Ease of fabrication	Multi-stage deposition/annealing required to achieve thicker films	Single step	Single step
Back-end CMOS compatible	No, exceeds thermal budget (>450 °C)	Yes	Yes
Highest reported refractive index	2.2 [45]	3.1 [52]	2.5 [65]
Highest reported nonlinear parameter, Kerr nonlinearity	6 W^−1^ m^−1^, 1.1 × 10^−18^ m^2^/W [45]	550 W^−1^ m^−1^, 2.8 × 10^−17^ m^2^/W [52]	95 W^−1^ m^−1^, 9.8 × 10^−18^ m^2^/W [65]
Film stress (deposited thickness)	∼1 GPa (N.D.) [60]	316 MPa (255 nm) [48]	191 MPa (568 nm) [69]

N.D., no data.

**Table 2:** List of SiN:D works presenting MRR frequency combs including their optical properties, comb types and pump schemes.

Waveguide core	Propagation	Quality factor	Pumping	Pump power	Comb type	Work,
dimensions (W × H)	loss		configuration			year
2.3 µm × 0.92 µm	0.31 dB/cm	1.6 × 10^6^, loaded	Single pump	440 mW	Modulational instability	[69], 2018
2.0 µm × 0.88 µm	0.17 dB/cm	2.29 × 10^6^, loaded	Single pump	100 mW	Perfect soliton crystals	[67], 2021
1.8 µm × 1.0 µm	Not disclosed	0.8 × 10^6^, loaded	Auxiliary assisted	400 mW (Aux)	Single soliton	[75], 2022
				500 mW (main)		
2.2 µm × 0.85 µm	0.09 dB/cm (TE)	0.5 × 10^6^, loaded (TM)	Auxiliary assisted	190 mW (Aux)	Single soliton	[70], 2022
		0.65 × 10^6^, intrinsic (TM)				
				<220 mW (main)		
		1.5 × 10^6^, loaded (TE)				
		2.0 × 10^6^, intrinsic (TE)				

**Table 3:** SiNx:D works reported in literature for integrated optics including process conditions and optical properties of films and devices.

*n* @	Gas flow rates	Pressure	Temp	RF	ICP	CVD type	Waveguide core	Propagation	Resonator quality	Work, year
1550 nm				power	power	CVD type	dimensions (W × H)	loss @ 1550 nm	factor	
1.87	40 sccm SiD4, 10 sccm Ar, 100 sccm N2	N.D.	200 °C	N.D.	N.D.	ECR-PECVD	1.1 µm × 0.55 µm	0.47 dB/cm	Not applicable	[56], 2017
1.98	36 sccm SiD4, 31 sccm N2	10 mTorr	270 °C	0 W	1000 W	ICPCVD	2.3 µm × 0.92 µm	0.31 dB/cm	1.6 × 10^6^, loaded	[69], 2018
1.99	N.D.	N.D.	270 °C	N.D.	N.D.	ICPCVD	2.0 µm × 0.88 µm	0.17 dB/cm	2.29 × 10^6^, loaded	[67], 2021
1.95	N.D.	N.D.	250 °C	N.D.	N.D.	ICPCVD	6.0 µm × 80 nm	2.23 dB/m (TE) 2.04 dB/m (TM)	11.18 × 10^6^, loaded (TE) 4.16 × 10^6^, loaded (TM)	[68], 2022
1.95	N.D.	N.D.	200 °C	N.D.	–	ECR-PECVD	1.8 µm × 1.0 µm	Not disclosed	0.8 × 10^6^, loaded	[75], 2022
1.96	N.D.	N.D.	270 °C	N.D.	–	ICPCVD	2.2 µm × 0.85 µm	0.09 dB/cm (TE)	0.5 × 10^6^, loaded (TM) 0.65 × 10^6^, intrinsic (TM) 1.5 × 10^6^, loaded (TE) 2.0 × 10^6^, intrinsic (TE)	[70], 2022
2.46	N.D. SiD4:N2 ratio 3:250	N.D.	350 °C	N.D.	–	PECVD	5.0 µm × 0.31 µm	1.5 dB/cm	6.2 × 10^4^, loaded (TE) 6.47 × 10^4^, intrinsic (TE)	[65], 2022
2.34	N.D. SiD4:N2 ratio 2:250	N.D.	350 °C	N.D.	–	PECVD	2.0 µm × 0.35 µm	N.D.	1.27 × 10^5^, intrinsic (TE)	[76], 2022
2.52	N.D. SiD4:N2 ratio 4:250	N.D.	350 °C	N.D.	–	PECVD	2.0 µm × 0.32 µm	N.D.	1.18 × 10^5^, intrinsic (TM)	[76], 2022

N.D., not disclosed.

**Table 4:** Optical properties of SRN:D films reported in literature.

Refractive	SiD_4_:N_2_ gas	Optical	Work, year
index	ratio	bandgap	
2.34	2:250	2.02 eV	[76], 2022
2.46	3:250	1.94 eV	[65], 2022
2.52	4:250	1.92 eV	[76], 2022

The aforementioned work reaped the benefits of a deuterated SiO_2_ cladding but did not fully take advantage of the deuterated process for the silicon nitride waveguide core. More recently, silicon nitride waveguides (cross section 6 µm × 80 nm) were fabricated with deuterated silane for both the silicon nitride core and SiO_2_ cladding, yielding waveguides with losses as low as 2 dB/m and ring resonators with a loaded quality factor *Q*_*L*_ of 11.2 million (Figure 3(c)) for the quasi-TE mode at 1550 nm [68]. In the same work from Bose et al., the performance of the SiN:D/SiO_2_:D devices were compared to hybrid SiN devices. These hybrid devices were fabricated using an 800 °C LPCVD-deposited SiN:H core and 250 °C PECVD-deposited SiO_2_:D cladding, and comparisons in performance showed a small improvement to 1.57 dB/m but a six-fold decrease in the *Q*_L_ to 1.98 million (Figure 3(d)). When using 800 °C LPCVD SiN annealed for 7 h at 1050 °C with deuterated SiO_2_ cladding, the waveguides outperformed the SiN:D/SiO_2_:D devices, attaining losses of 1.00 dB/m and a *Q*_L_ of 25.5 million (Figure 3(e)). This study provides a comparison of the losses in the various core and cladding materials and shows that deuterated silane provides an avenue for ultra-low-loss waveguides, on the order of dB/m, which is sufficient for most applications.

While the nonlinear parameters of these propagating modes are inevitably lower due to low modal confinement and are thus not ideal for nonlinear optical applications, these devices are prime candidates for applications requiring very low losses such as use as waveguide interconnects for information carriers, gyroscopes and optical reference cavities [77]. These works ultimately show that the substitution of hydrogen for deuterium in the deposition of silicon nitride films makes for a highly effective single-step deposition process for the creation of very low-loss, back-end CMOS-compatible integrated optical devices.

## 3 Recent work in nonlinear optics

Apart from generating low-loss waveguides, SiN:D is currently being studied with great interest for nonlinear optics. This endeavour differs from the fabrication of ultra-low-loss devices in the previous section, which focused on the design goal of the lowest possible losses, a key approach being the exploitation of weakly confined waveguide modes using a thin waveguide core. In nonlinear optics applications, low losses are important, but not the only variable in the equation. Both the Kerr nonlinearity (*n*_2_) of the waveguide and the modal profile play a strong role in the resulting efficiency of nonlinear phenomena. Nonlinear dynamics in photonic devices may be described using the nonlinear Schrödinger equation [78]:

(3) $$\frac{\partial A}{\partial z}=-\frac{\alpha }{2}A+i\overset{\infty }{\underset{k=2}{\sum }}\frac{i^{k}}{k!}\beta_{k}\frac{\partial^{k}A}{\partial T^{k}}+i\gamma \left({\left|A\right|}^{2}A\right)$$

where *z* is the longitudinal coordinate and *α* is the linear loss coefficient. *A*, T and *β*_k_ are the slow varying pulse envelope, time and dispersion of *k*th order, respectively. The nonlinear parameter, $\gamma =\frac{2\pi n_{2}}{\lambda A_{\text{eff}}}$ , where *λ* is the wavelength, and *A*_eff_ the effective modal area of the propagating mode. *γ* in particular is a strong contributor to many nonlinear effects leveraging the 3rd order nonlinearity of a material, including Four-Wave Mixing (FWM), broadband frequency comb and supercontinuum generation, self-phase modulation and optical amplification. In resonant structures such as the prolific microring used for frequency comb generation, nonlinear dynamics may be described using the Lugiato–Lefever equation, interpreted as the damped and driven nonlinear Schrodinger equation [79]:

(4) $$\begin{array}{ll} T_{R}\frac{\partial }{\partial T}A & =-\left(\frac{\alpha }{2}+i\delta \right)A+i\underset{k>1}{\sum }\frac{\beta_{k}}{k!}{\left(i\frac{\partial }{\partial t}\right)}^{k}A\\ & \;-j\gamma {\left|A\right|}^{2}A+\sqrt{\theta }A_{in} \end{array}$$

where *A* is the envelope of the electric field, *β*_k_ describes the dispersion of *k*th order, *T*_R_ is the round-trip time in the microresonator, *α* is the round-trip loss, *T* and *t* are the slow and fast times respectively, *δ* is the detuning between pump and closest resonance, *θ* is the resonator-waveguide coupling coefficient and *A*_in_ is the continuous wave input. The equation accounts for the driving effect from the pump and the damping effect from dispersion and may be viewed as a modified version of the nonlinear Schrödinger equation used to describe nonlinear pulse propagation, with a key difference being the driving term represented by *A*_in_.

As may be observed from both Eqs. (3) and (4), that *γ* plays an important role in the nonlinear optical phenomena. A larger nonlinear parameter lowers the amount of power required to observe waveguide-based nonlinear optics such as supercontinuum generation. In resonant-enhanced nonlinear optics, it similarly enables a reduction in the threshold power required for frequency comb generation, an area of research that currently has significant momentum in the field. For a fixed wavelength, improvements in the nonlinear parameter can be achieved by either (1) increasing the Kerr nonlinearity of a material or (2) decreasing the effective modal area of the propagating mode. In the case of SiNx devices, both of these occur when the silicon content in the films is increased, which can be engineered by increasing the ratio of silicon to nitrogen carrier precursor gases during CVD.

### 3.1 SiN:D Kerr frequency combs

The utility that frequency combs (FCs) have provided for the field of optics is indisputable, seeing use in applications such as high precision metrology [80], spectroscopy [81] and optical sources [21, 82]. Originally associated with the generation of mode-locked lasers, the generation of Kerr-microcombs in minute optical cavities such as Whispering Gallery Mode (WGM) resonators and planar-integrated MRRs has been a dominating branch of research in the community for the past 15 years. The first demonstration of Kerr frequency combs in a microscopic optical cavity was presented in 2007 [83]. Since then, significant progress has been made in microresonator-based Kerr frequency combs, which are planar in nature [11, 84–86].

Of intense interest is the generation of Dissipative Kerr Solitons (DKSs) [13, 19, 87] and the formation of temporally ordered micro-resonator soliton crystals [20, 88] – coherent, self-reinforcing pulses of light that result from a balance between dispersion and nonlinearity – in planar-integrated microcavity devices, which possess immense potential in space-sensitive applications such as portable light detection and ranging (LIDAR) devices [89]. Advancements in portability and miniaturisation have also seen these devices go from requiring the use of extensive table-top experimental setups to battery-powered comb generators [90], and SiN:D devices stand to make these technologies more easily accessible given their ease of fabrication and desirable optical properties.

Advancements in SiN:D technologies have largely followed this trend in micro-resonator research – in recent works, SiN:D devices have been frequently employed in the generation of broadband frequency combs (Table 2), with successive works improving in losses and exhibiting different comb characteristics. Modulational instability (MI) frequency combs in SiN:D MRRs were first demonstrated by Chiles et al. in 2018 [69]. In this work, the films were deposited using ICPCVD at a substrate temperature of 270 °C and chamber pressure of 10 mTorr, using SiD4 and N_2_, resulting in 860 nm thick layers and a series of ring resonators realised using electron-beam lithography and an ICP-based reactive ion etching process. The resonators possessed waveguide cross sections of 1.5/1.6/2.1 µm × 0.86 µm (width × height) and identical ring radii of 23 µm, featuring quality factors as high as 1.6 × 10^6^. Notably, the films were characterized to have low tensile stress (191 MPa for 568 nm thick films). Conversely, LPCVD grown SiN films typically have high stress on the order of 1 GPa [48, 60], which necessitates the use of stress management techniques such as multi-step deposition methods used in the photonic damascene process or proper definition of trenches homogenously throughout the entire wafer in order to achieve thick films that are required to achieve anomalous dispersion. This is in addition to the very high thermal budgets required of up to 1200 °C, where most processes use a 2- to 3-hour anneal time and some perform annealing after each of the multi-stage deposition steps. In contrast, films required for SiN:D devices possessing anomalous dispersion can be deposited in a single-step deposition process without the need for additional annealing, which has the additional benefit of preserving back-end CMOS compatibility.

**Figure 4:**
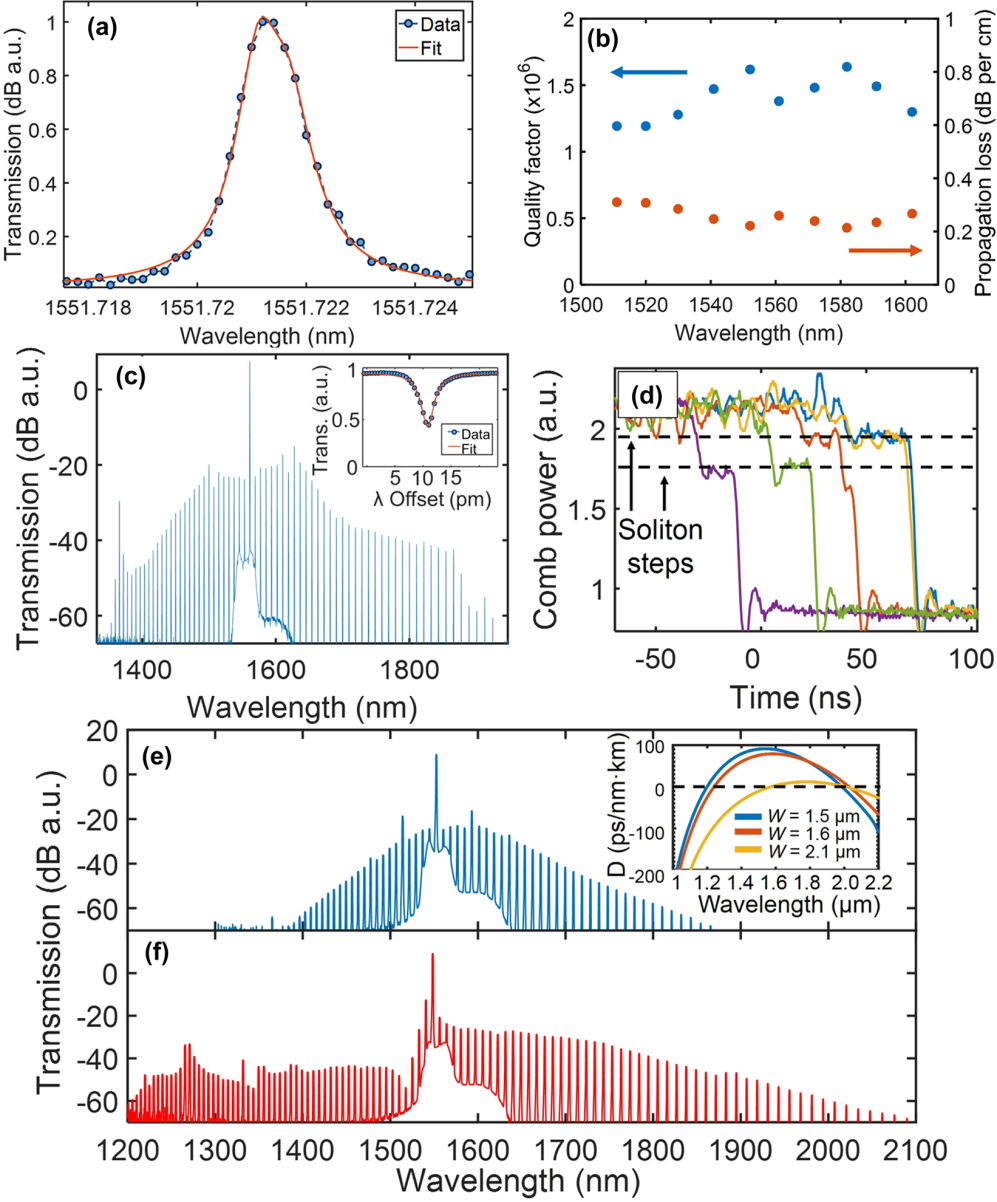
Optical characterization of a microring resonator fabricated using deuterated silicon nitride. (a) Transmission spectrum of the quasi-TE resonant mode at the drop port located at 1552 nm. (b) The measured quality factor and corresponding propagation loss as a function of wavelength. (c) Frequency comb spectrum generated from a microresonator with a width and height of 1.5 μm and 900 nm, respectively. The inset shows the resonant mode used to generate this comb with loaded *Q* of 5.6 × 10^5^. (d) Measured comb power (using an oscilloscope) during repeated red detuning of the pump laser frequency, showing evidence for dissipative Kerr-soliton transient formation with two discrete and repeatable step levels. The generated frequency comb for ring widths of (e) 1.6 and (f) 2.1 μm. The inset in (e) shows the numerically calculated dispersion for the zeroth order TE mode for widths of 1.5, 1.6 and 2.1 μm. Adapted from [69].

The MI comb spectra was achieved at a coupled pump power of 440 mW, obtaining a wide spectrum spanning 1350 nm to 1900 nm ring widths of 1.5 µm and 1.6 µm while the resonator with a ring width of 2.1 µm exhibited comb spectra spanning 1200 nm–2100 nm. Importantly, the considerably wider generated comb spectrum for a ring width of 2.1 µm may be attributed small dispersion magnitude (inset of Figure 4(e)) over a wide bandwidth, facilitating efficient phase matching. While soliton steps were observed in the measured spectra (Figure 4(d)), stable soliton generation was not achieved in this work, with the authors citing strong thermal shifts in the resonance due to a larger absorption coefficient compared to annealed LPCVD SiN films. It is also worth noting that the already low propagation losses of 0.31 dB/cm are observed to be the highest recorded propagation loss values for SiN:D devices employed in the generation of frequency combs thus far.

**Figure 5:**
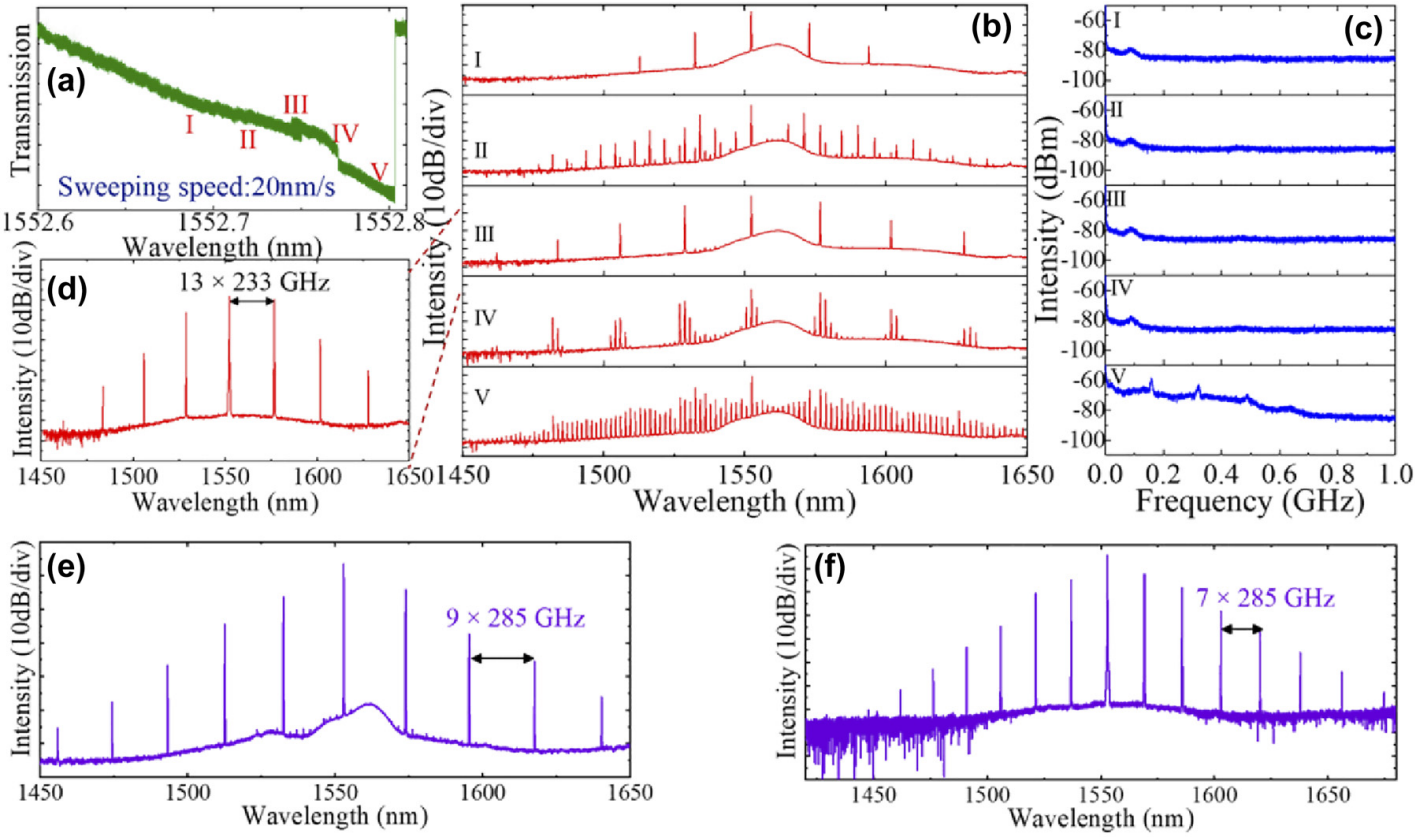
Experimental results of Turing and Perfect Soliton Comb (PSC) states realised on an SiN:D microresonator. (a) Pump transmission as a function of 100 mW for a SiN:D microring resonator with 100 µm radius and on-chip power of ∼100 mW. (b) Frequency comb states and (c) intensity noise corresponding to the wavelengths denoted in (a). (d) Soliton crystal state III with ASE noise filtered out (zoomed in), prior to coupling into the waveguide. Spectral trace of (e) 9-PSC and (f) 7-PSC soliton combs [67].

Since MI combs in SiN:D MRRs were demonstrated by Chiles et al., frequency combs generated on the SiN:D platform have seen improvements in required pump power and comb coherence. Using SiN:D films grown using ICPCVD at a low temperature of 270 °C, Wu et al. [67] demonstrated MRRs with losses as low as 0.17 dB/cm and a loaded quality factor of 0.95 million. They also demonstrated microdisk resonators with a loaded quality factor of 0.7 million on the same platform [71]. Using SiN:D MRRs of various designs, perfect soliton crystals requiring coupled pump powers of only 13.5 mW for optical parametric oscillation and 100 mW for comb generation were demonstrated. Figure 5(a) shows the transitions from the Turing state (I) to the modulation instability state (II, IV and V), which is typically incoherent and noisy. The generated frequency combs corresponding to each of these detuning values are shown in Figure 5(b) and (d). The detuning corresponding to state III generates a soliton crystal state shown in Figure 5(c) to possess low RF noise. Soliton crystals were generated by driving a single pump laser into the blue-detuned regions of the resonance with a sweeping rate of 20 nm/s, attaining 7-PSC and 9-PSC results (combs with spacings 7 times and 9 times the free-spectral range) in different devices (Figure 5(e) and (f)). However, while the soliton crystal states can be stably accessed with a number of different devices, the stability of the crystal does seem to be intermittent given that they break with other reported soliton crystal dynamics. The crystal states are shown to revert to incoherent MI comb states with further pump detuning, diverging from previously reported dynamics observed in simulations using the Lugiato–Lefever equation where solutions leading to PSC comb generation lies beyond the MI comb region [20].

**Figure 6:**
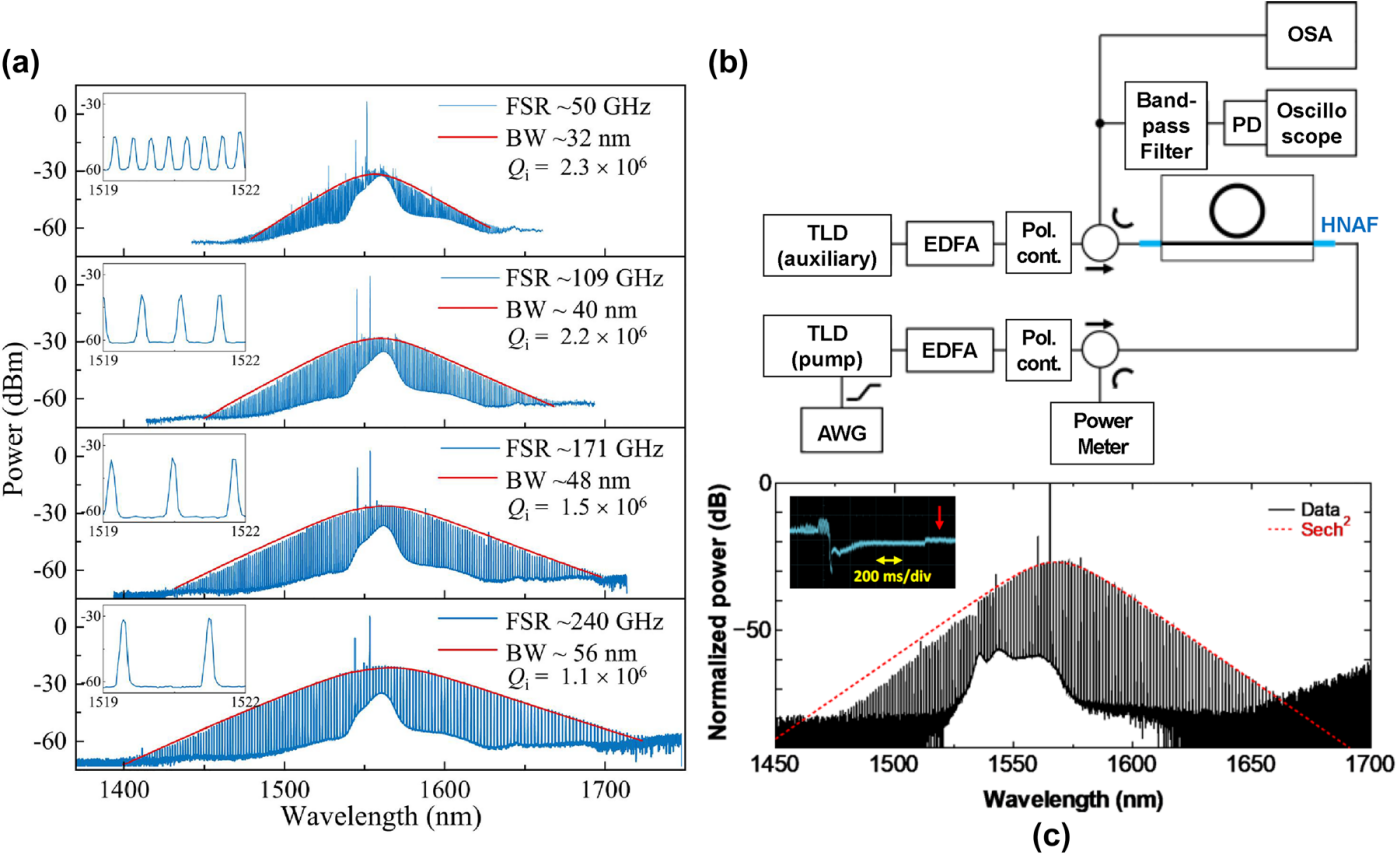
Single-soliton generation in SiN:D MRRs with different free spectral ranges. Figures show measured optical spectra 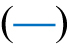 and sech^2^ fit 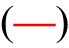 characteristic of singly generated DKSs in MRRs of different radii, corresponding to different FSRs observed in the plot. This experimentally confirms that the platform is stable enough to facilitate single-soliton generation, a feature not seen in PECVD SiN:H MRRs thus far due to high material absorption near 1.55 µm as a result of parasitic hydrogen bonds. Consistent presence of two peaks in the spectrum arises from a dual-pump scheme described in [54] that suppresses relatively slower thermal dragging effects, relaxing the strict constraints of the scan speed required in previous single-pump schemes. Figure adapted from [70]. (b) Auxiliary laser setup used to generate the single-soliton comb state and (c) measured optical spectrum. Inset shows transmittance during laser wavelength sweep. (b) and (c) adapted from [75].

Most recently in 2022, SiN:D MRRs have also been employed in the generation of highly coherent single-soliton frequency combs [70, 75]. Figure 6 shows the experimental setup and sech^2^ spectra associated with single-soliton generation from [70] (Figure 6(a)) and [75] (Figure 6(b) and (c)), respectively, achieved with ring resonators of various ring radii. Notably, both works achieved single-soliton generation with the use of auxiliary lasers as described in [91], which differs from prior experimental schemes for soliton generation (single soliton or otherwise) [13, 92, 93] where a single pump laser was employed. DKSs are then generated by avoiding delayed thermo-optic effects through a judicious selection of the laser sweep rate. The auxiliary pump scheme is designed to mitigate these thermal dragging effects by coupling an auxiliary laser into another resonance, which relaxes the sweeping conditions required for the realisation of solitons by keeping cavity temperature relatively unchanged when the pump laser is shifted to the red-detuned region of the resonance. This setup is significantly more complicated and may speak towards the higher thermal susceptibility compared to LPCVD SiNx:H that was previously reported in [69]. Nonetheless, generating these solitons also usually require devices that have low thermal susceptibility such that mode locking can be sustained, which meant that these nonlinear phenomena were not previously privy to PECVD SiNx:H films that featured higher hydrogen concentrations and absorption losses.

The successful realisation of PSCs and DKSs on the SiN:D marks its promise as a simple, low-loss, back-end CMOS-compatible material platform nonlinear optical applications and unlocks nonlinear phenomena previously available only to high temperature annealed LPCVD SiN:H films. LPCVD SiN:H MRRs presently outperform SiN:D MRRs in terms of loss performance – the demonstration of octave-spanning frequency combs in SiN:H MRRs in 2011 [12] employed an MRR with a quality factor of 3 million, outperforming the present state-of-the-art SiN:D MRR at 2.3 million. In general, the high confinement resonators (which frequency combs fall under to meet anomalous dispersion requirements) fabricated on annealed LPCVD SiN:H have quality factors on the order of 10^6^–10^7^ [94, 95], though these authors have also stated that they have spent many years developing detailed process refinements to achieve such low losses. SiN:D is an emerging field, nascent compared to LPCVD SiN:H for photonics where the first papers on it were published in 2011 and is inevitably much more technologically mature. The fact that the best SiN:D resonators today are only about 10 times smaller in quality factor compared to the much more established LPCVD SiN:H shows that with time and the relevant process improvements, further increases in quality factor could be on the horizon (Table 3). In addition, the reduction in fabrication complexity and the potential of integrating BEOL processes with SiNx:D provides a promising outlook, and future improvements to fabrication processes might bring the loss performance of SiN:D devices down even further.

### 3.2 SiNx:D Kerr nonlinear optics

The nonlinear domain in integrated optics is usually accessible with a sufficiently high combination of light intensity and device nonlinearity. For example, optical parametric oscillations (OPOs) require a minimum threshold power before they are observed, and in fundamental 3rd order nonlinear effects such as Four-Wave Mixing (FWM) and Self-Phase Modulation (SPM), the nonlinear efficiency increases with the Kerr nonlinearity of the medium. When considering the integration of technologies into real-world systems, however, power consumption remains a core consideration due to concerns regarding cost and portability, which drives a never-ending pursuit for more efficient systems.

In the case of silicon nitride, the manipulation of the Si:N ratio of the film can lead to a variation in its physical and optical properties including film stress, photoluminescence refractive index amongst others [53, 96, 97]. Most importantly for nonlinear optics, an increase in silicon content also contributes to an increase in refractive index, which increases the nonlinearity of the film by virtue of Miller’s rule [98–100]:

(5) $$n_{0}={\left(1+\chi^{\left(1\right)}\right)}^{\frac{1}{2}}$$

(6) $$n_{2}=\frac{3}{8n_{0}}\chi^{\left(3\right)}$$

(7) $$\chi^{\left(3\right)}\propto {\left(\chi^{\left(1\right)}\right)}^{4}$$

Here, *n*_0_ is the linear refractive index, *χ* ^(*i*)^ is the *i*-th order electric susceptibility of the waveguiding media and *n*_2_ is the material Kerr nonlinearity. The increase of *n*_2_ in turn leads to an equivalent improvement of the nonlinearity experienced by the propagating mode within the waveguide that results in greater efficiency in the generation of nonlinear effects.

In CVD-deposited SiNx, the silicon content of the film can be easily tuned by changing the flow rates of relevant precursor gases within the chamber and by tuning the deposition parameters to produce high quality films. In recent years, conventional silicon-rich nitride (SRN:H) based nonlinear optics has become a popular field of study, spawning a number of works leveraging both LPCVD [39, 45, 101] and PECVD [44, 47, 102], including the demonstration of high spectro-temporal compression [103], broadband parametric amplifiers [102] as well as supercontinuum generation [52, 101] and high-speed data processing in the nonlinear regime [46].

Demonstrated applications of SRN:H stand to benefit from the use of SRN:D due to the back-end CMOS-compatible fabrication process and reduced loss when compared to PECVD SRN:H. Although LPCVD SRN:H has been observed contain lower losses compared to their PECVD counterparts, reported refractive indexes also tend to be much lower despite high DCS:N_2_ gas ratios, hovering around 2.2 ≤n≤ 2.3 at the higher end [39]. By virtue of Eqs. (5)–(7), this lower refractive index has also translated to much lower values of the nonlinear parameter *γ* observed in LPCVD devices (*n* = 2.2, *γ* = 6 W^−1^ m^−1^, *n*_2_ = 1.4 × 10^−18^ m^2^/W [45]) as opposed to PECVD-based SRN:H (*n* = 3.1, *γ* = 550 W^−1^ m^−1^, *n*_2_ = 2.8 × 10^−17^ [47]). The limits on the refractive index seem to be contingent on the type of deposition employed and not the precursor gases, as SRN:H devices deposited using DCS and N_2_ gas have also been demonstrated to achieve a refractive index close to 3.1 [51].

Given the discussed shortcomings of that exist for LPCVD and PECVD SRN:H devices, one can see that there exists a need for a back-end CMOS-compatible material with high nonlinearity and low material absorption losses, which can be filled by high-quality SRN:D films. SRN:D devices have been demonstrated to retain the same tuneable film characteristics as has been observed in SRN:H; namely, the PECVD-deposited films are easily tuneable by changing the precursor gas ratios within the chamber. To date, SRN:D films have been realised with refractive indexes ranging from 2.34 to 2.52, using SiD_4_:N_2_ gas ratios ranging from 1:125 to 2:125 [65, 76, 104] (Table 4). Predictions by Miller’s rule have also held true for linear optical parameters where increased refractive indexes and lowered optical bandgap of the films correlated with the input gas ratios.

**Figure 7:**
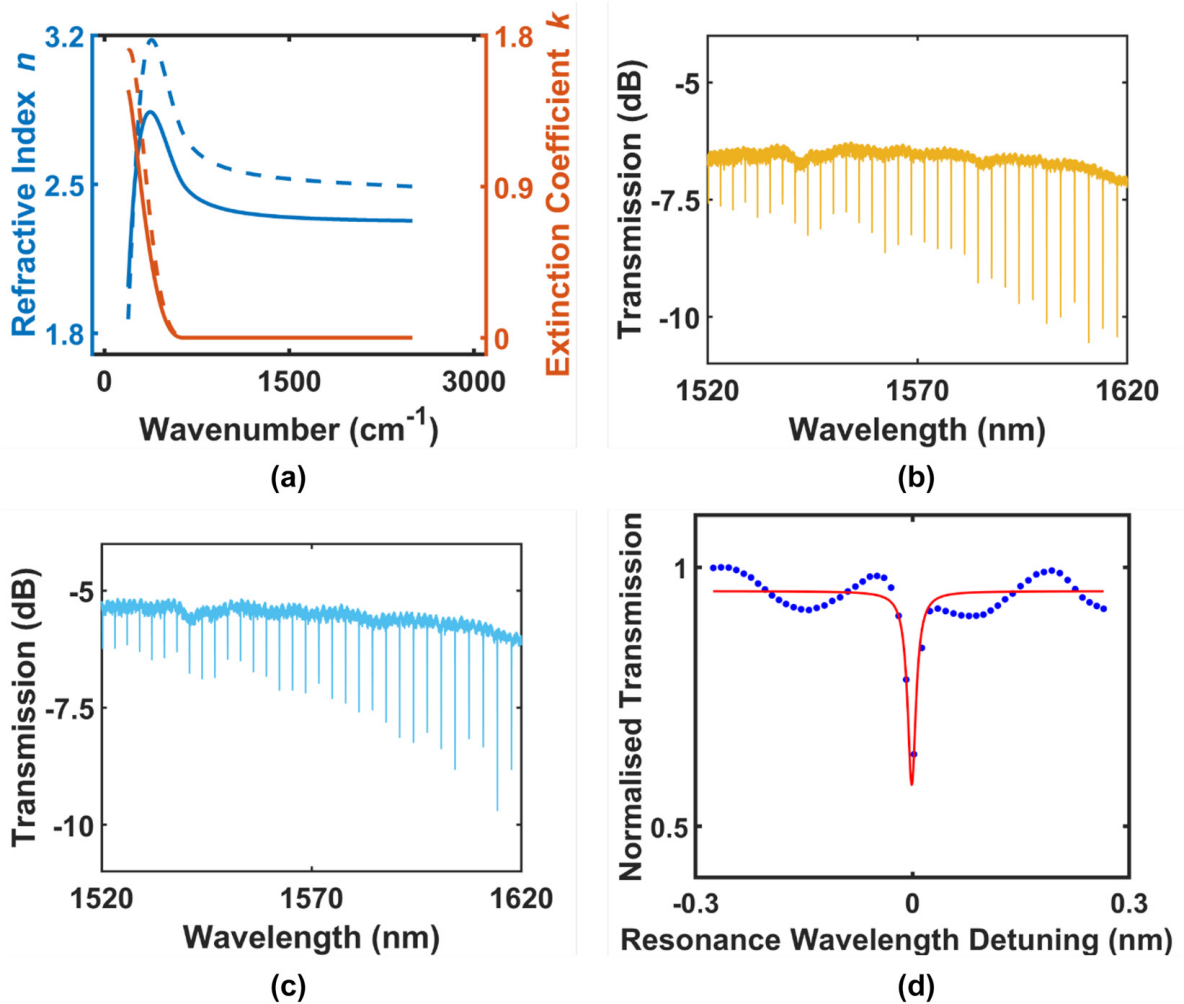
Characterisation results from SRN:D films and fabricated microresonators. (a) Refractive index (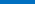) and nonlinear coefficient (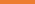) data of film A (solid lines) and film B (dotted lines) determined using FTIR spectroscopic ellipsometry, (b) measured TE mode transmission of an MRR on the SRN:D film with refractive index *n* = 2.34 and (c) measured TM mode transmission spectrum of an MRR on the film with *n* = 2.52. Both resonators have a ring radius of 50 μm and ring width of 1 μm, (d) Lorentzian fit (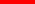) and experimentally measured transmission spectrum (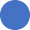) of a characteristic dip in the transmission spectrum of a ring resonator with an intrinsic quality factor of 127,000 in TE mode. Figure adapted from [76].

**Figure 8:**
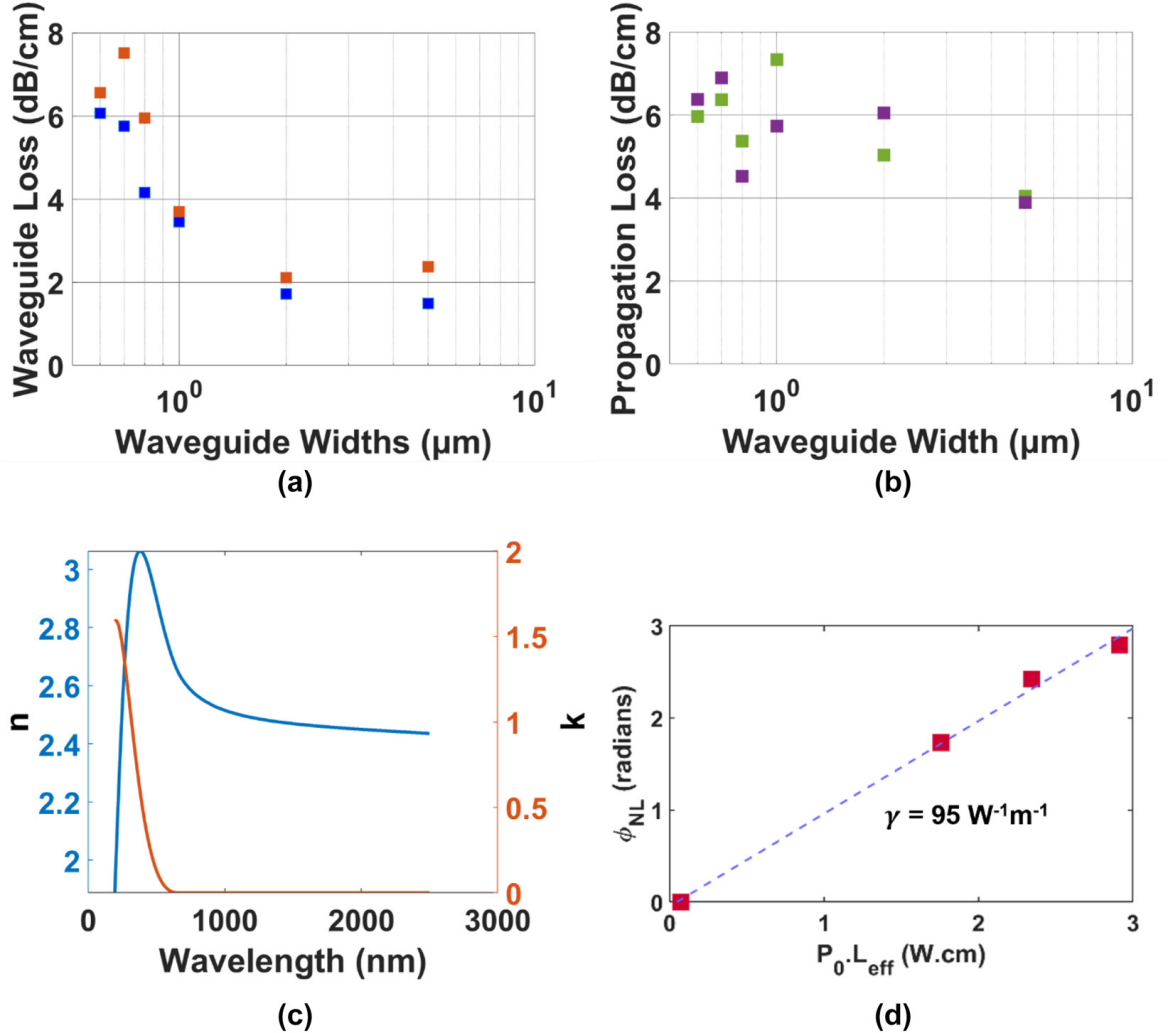
Characterisation results of waveguides fabricated on an SRN:D film with a refractive index of n = 2.46 at 1550 nm. (a) Waveguide propagation losses from cutback measurements on an SRN:D platform deposited with a SiD_4_:N_2_ gas ratio of 3:250 for TE (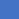) and TM (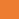) modes (b) and on an SRN:H platform (fabricated using SiH4) for TE (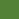) and TM (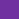) modes. Recipe used for SRN:H deposition is identical to that of Figure 8(a). (c) Refractive index (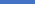) and extinction coefficient (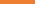) of the PECVD SRN:D film, determined using spectroscopic ellipsometry, showing a linear refractive index of 2.46 at 1.55 µm. (d) Nonlinear parameter fit (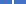) of the experimentally determined nonlinear phase shift (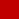) observed in self-phase modulation experiments across different pump powers. The extracted nonlinear parameter is 95 W^−1^ m^−1^. Figure adapted from [65].

Presently, the dominant source of losses in SRN:D devices is observed to arise from sidewall roughness [65], which is evidenced by the exponentially decreasing loss performance of fabricated waveguides, ranging from 8 dB/cm to 1.5 dB/cm with increasing waveguide widths (Figure 8(a)). When compared to SRN:H devices fabricated using identical fabrication techniques, the benefits of using SiD_4_ are easily observable in the ∼2 dB/cm loss improvement at wider waveguide widths, and the contribution of parasitic hydrogen bonds in SRN:H that lead to an erratic variation of the waveguide propagation losses (Figure 8(b)). MRRs with intrinsic quality factor as high as 127,000 in the TE_00_ mode (Figure 7(d)) and 118,000 in the TM_00_ mode have also been achieved, which are fairly comparable to LPCVD SRN:H MRRs in literature despite the higher achieved refractive index of the SRN:D film [39]. The latent 1.5 dB/cm loss within the devices could possibly be attributed to silicon clusters within the film that act as scattering centres for propagating light. These clusters have been observed in silicon-rich films [39, 45, 53] and can greatly hamper device performance in the linear regime by acting as scattering centres or as two-photon absorption sources in the presence of high intensity light.

SRN:D films and devices have also been characterised for their nonlinear optical properties [65]. In this work, the platform was fabricated using a precursor gas ratio of 3:250 (SiD_4_:N_2_) and was measured to have a linear refractive index of 2.46. Devices fabricated were observed to possess a nonlinear parameter *γ* = 95 W^−1^ m^−1^ using self-phase modulation experiments, where the nonlinear phase shift of input pulses was recorded and plotted against the peak input power *P*_0_ (Figure 8(d)). Subsequent simulations of the modal area allowed for the calculation of the film Kerr nonlinearity of *n*_2_ = 9.8 × 10^−18^. This is roughly 35 times as high as stoichiometric SiN devices, and the films are confirmed to retain an optical bandgap above the two-photon absorption threshold of 1.6 eV. Propagation losses determined using cutback measurements are 2–2.5 dB/cm lower compared to reference SRN:H devices that were prepared using the same recipe but with SiH_4_ gas instead of SiD_4_ gas.

The increased nonlinearity of SRN:D films offer the potential of enhanced nonlinear effects over current SiN:D devices. As noted in Section 3.1, frequency combs and MRR-generated solitons from a continuous-wave input are research areas that are presently in high demand, not least because of the widespread potential that these devices possess. Thus far, however, the demonstration of frequency combs in SRN:H has been quite elusive, with only a handful of works successfully demonstrating frequency combs on platforms with a Kerr nonlinearity 3–4 times above stoichiometric SiN [36, 105, 106]. Notably, these films are fabricated with LPCVD and reactive sputtering, with little mention of PECVD-deposited devices due to the high hydrogen content that makes it difficult to lock resonances in SRN:H MRRs. Current successes in SiN:D soliton combs and high-quality factor SRN:D MRRs should be seen as promising signs for demonstrations of soliton microcombs in highly nonlinear SRN:D devices in the near future.

## 4 Conclusion and future outlook

Today’s state of the art low-loss nonlinear photonic devices in silicon nitride are largely fabricated using high temperature LPCVD followed by several hours of annealing at temperatures of up to 1200 °C. In certain cases, multiple wing steps are adopted [107]. The high temperatures used aim to eliminate Si–H bonds and their associated absorption. This same phenomenon – that using deuterated silane in place of regular silane eliminates – may be achieved at significantly lower temperature (<400 °C) chemical vapour deposition processes, potentially making ultra-low-loss SiNx available as a back-end CMOS-compatible process. Consequently, demonstrations of SiNx:D PICs have increased in frequency since their emergence and stand to become more common as they are leveraged for their low-loss, CMOS-compatible properties, their nonlinear functionalities potentially extended by increasing the silicon content to achieve silicon-rich films.

Further advancements in the platform could yield even lower losses. For example, the loss of high-confinement waveguides and MRRs fabricated on the platform stand to be improved through the use of an SiO_2_:D cladding instead of conventional SiO_2_, which will push the propagation loss lower than 0.1 dB/cm observed in Ref. [70]. Improvements in device fabrication in SRN:D films such as [65] could also greatly lower the susceptibility of Si-rich waveguides towards scattering losses due to sidewall roughness. Deposition methods and process refinements that reduce the incidence of Si nanocluster formation could also be studied to improve the quality of films. The back-end CMOS compatibility of the material opens up opportunities for electrical integration such as the placement of microheaters for on-chip thermo-optic applications [108] that were previously inefficient with SRN:H devices.

One important potential implication of high-quality, low-loss, low-temperature SiNx:D platforms is their more straightforward, seamless integration with on-chip systems, which also require application specific integrated circuits (ASICs). In these systems, such as silicon photonics based [109–111], the typical process flow requires ASICs to be defined on the front end (high thermal budget), whereas photonics are defined on the back-end, which has a low thermal budget typically below 450 °C. For frequency comb-based devices to become mainstream and applicable to a wide swath of applications, not limited to data centre communications and telecommunications, lower thermal budgets than those required in LPCVD-based silicon nitride devices are required. SiNx:D could play an instrumental role in filling this gap, enabling efficient nonlinear optical functions to become more widely adopted.

Currently, demonstrated applications in SiNx:D have largely concentrated around the realisation of broadband nonlinear effects such as frequency combs and supercontinuum generation, as well as soliton generation. With the significant progress made in the SiNx:D platform to date, the development of other important nonlinear optics functions including high gain parametric amplifiers and ultrafast optical switches could be on the horizon. Nonlinear devices using SiNx:D, for example, would perform significantly better than other BEOL CMOS-compatible alternatives such as crystalline or amorphous silicon, which possess two-photon and free-carrier absorption, limiting their temporal response or achievable gain in the case of parametric amplifiers. With further development, their use could be extended to wider domains, which leverage nonlinear optical phenomena such as quantum information processing and microwave photonics. Lastly, the vast majority of work on SiNx:D thus far has focused on the Kerr effect. However, studies into other nonlinear optical effects such as its Raman response [72], potential for Brillouin scattering [112] and potentially presence (or inducement) of a χ^(2)^ response [113] could reveal even greater utility for the SiNx:D platform for nonlinear optics.

In conclusion, the SiNx:D platform has been shown to be effective in creating low-loss, low thermal budget devices with good and efficient demonstrations of nonlinear optics functions thus far. Its strong potential to provide an alternative to high thermal budget devices used today in a plethora of nonlinear optics functions is of great importance and its development timely. Future developments in both the SiNx:D platform and devices developed from it could yield new innovations, importantly not constrained by process, as well as drive their proliferation into commercial applications.
